# Multiscale responses and recovery of soils to wildfire in a sagebrush steppe ecosystem

**DOI:** 10.1038/s41598-022-26849-w

**Published:** 2022-12-23

**Authors:** Kathleen A. Lohse, Derek Pierson, Nicholas R. Patton, Jonathan Sanderman, David P. Huber, Bruce Finney, Jeremy Facer, Jared Meyers, Mark S. Seyfried

**Affiliations:** 1 Department of Biological Sciences, Idaho State University, Pocatello, ID USA; 2 Department of Geosciences, Idaho State University, Pocatello, ID USA; 3 Woodwell Climate Research Center, Falmouth, MA USA; 4 Northwest Watershed Research Center, USDA ARS, Boise, ID USA; 5 Present Address: Division of Earth and Ecosystem Sciences, Desert Research Institute, Reno, NV USA; 6 Present Address: Department of Earth, Environmental and Resource Sciences, The University of Texas at El Paso, El Paso, TX USA; 7 Present Address: Rocky Mountain Research Station, USDA-Forest Service, Boise, ID USA

**Keywords:** Biogeochemistry, Fire ecology, Biogeochemistry

## Abstract

Ecological theory predicts a pulse disturbance results in loss of soil organic carbon and short-term respiration losses that exceed recovery of productivity in many ecosystems. However, fundamental uncertainties remain in our understanding of ecosystem recovery where spatiotemporal variation in structure and function are not adequately represented in conceptual models. Here we show that wildfire in sagebrush shrublands results in multiscale responses that vary with ecosystem properties, landscape position, and their interactions. Consistent with ecological theory, soil pH increased and soil organic carbon (SOC) decreased following fire. In contrast, SOC responses were slope aspect and shrub-microsite dependent, with a larger proportional decrease under previous shrubs on north-facing aspects compared to south-facing ones. In addition, respiratory losses from burned aspects were not significantly different than losses from unburned aspects. We also documented the novel formation of soil inorganic carbon (SIC) with wildfire that differed significantly with aspect and microsite scale. Whereas pH and SIC recovered within 37 months post-fire, SOC stocks remained reduced, especially on north-facing aspects. Spatially, SIC formation was paired with reduced respiration losses, presumably lower partial pressure of carbon dioxide (pCO_2_), and increased calcium availability, consistent with geochemical models of carbonate formation. Our findings highlight the formation of SIC after fire as a novel short-term sink of carbon in non-forested shrubland ecosystems. Resiliency in sagebrush shrublands may be more complex and integrated across ecosystem to landscape scales than predicted based on current theory.

## Introduction

**Figure 1:**
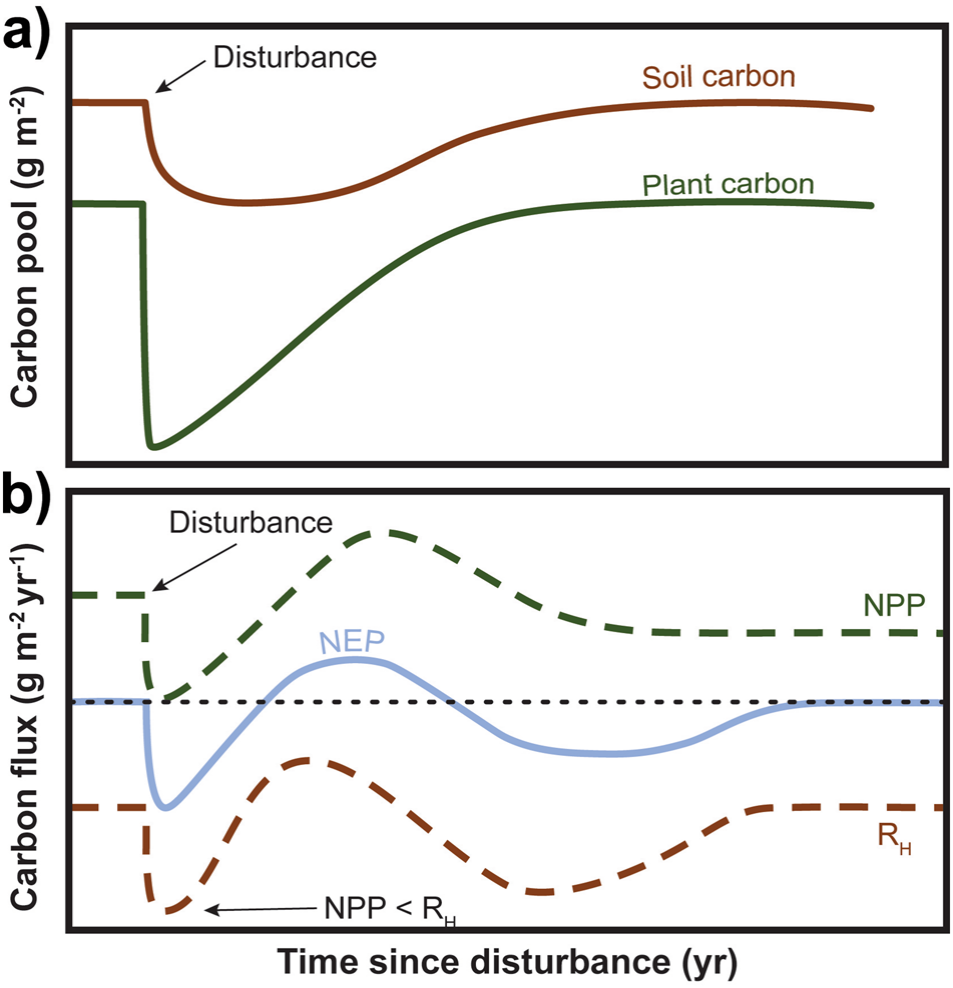
Predicted plant and soil carbon pools and processes following disturbance. Immediately after disturbance, loss of soil organic carbon and plant material are predicted (above panel). Prior to disturbance, net primary productivity (NPP) is in balance with hetetrophic respiration (R_H_) such that NEP equals zero. A pulse in respiration (R_H_) (bottom panel) is predicted after disturbance such that NPP < R_H_ and NEP is negative (source of carbon to the environment) and eventually the ecosystem comes back into quasi-steady state (modified from Gorham et al. ^2^).

Disturbance theory holds that a pulse disturbance, such as wildfire, will result in the loss of plant and soil organic carbon (SOC) via thermal combustion followed by a short period of elevated respiration (R_H_)^1,2^, These respiratory losses are predicted to exceed the recovery of net primary productivity (NPP) in the short term (i.e., R_H_ > NPP), such that net ecosystem production (NEP) is negative (Fig. 1). In recent years, this theoretical framework has been revisited^3^ and extended to incorporate pyrogenic carbon (PyC)^4^ and to consider directional press dynamics such as climate and vegetation change that may alter ecosystem development trajectories^5^. Despite these advancements, fundamental uncertainties remain in predicting recovery trajectories after disturbance where the spatial and temporal scales of ecosystem structure and function are not adequately represented. In particular, landscape scale processes may be especially relevant for understanding and improving disturbance trajectory models in heterogeneous, non-forested ecosystems such as sagebrush shrublands that are experiencing increased wildfire frequency and extent with climate change and proliferation of invasive grass species such as *Bromus tectorum* (cheatgrass)^6–8^.

Sagebrush steppe ecosystems cover much of the western United States (US) (165 million acres) and are characterized by their spatial variability in vegetation and soil at the ecosystem to landscape scale^9^. At the ecosystem (microsite) scale, the patchy distribution of shrubs results in higher concentrations of soil carbon, nitrogen, and water availability under plants compared to the spaces between the plants (interplant)^10,11^. At the landscape scale, vegetation, microbes, and soils shift systematically with elevation^12–15^ and topographic hillslope position^16^. In addition, hillslope aspect can result in pronounced differences in slope^17,18^, microclimate^19^, vegetation^18^, and soil carbon^13–15^. Seyfried et al.^19^, for example, showed that the mean annual soil temperature difference (5.2 °C) between a north- and southwest-facing aspects was greater in a 0.25 km^2^ catchment than the 4.4 °C difference associated with a 1000 m elevation change across an entire watershed (239 km^2^). Patton et al.^13^ also showed that north-facing aspects contained approximately 3 times more SOC than south-facing aspects. These landscape-level aspect effects may interact with or even trump ecosystem-scale shrub microsite effects to determine recovery trajectories following fire but these effects remain understudied.

Disturbance trajectory models have been proposed for shrublands that incorporate landscape-level properties (i.e. aspect and slope) to assess risk to disturbance as well as predict ecosystem recovery trajectories. In particular, Chambers et al.^20^ have posited high elevation and north-facing aspects to be more resistant to cheatgrass and also more resilient to disturbance compared to lower elevation and south-facing aspects. While numerous studies have focused on plant community responses to fire and generally confirmed lower resistance to cheatgrass invasion at lower elevations and on warmer south-facing aspects compared to north-facing ones^21,22^, comprehensive tests of aspect-driven recovery of soil properties and processes following fire remain lacking. Most studies have focused on ecosystem microsite (plant and interplant) responses within a year of a fire. In a meta-analysis of 39 studies from the Great Basin, USA, Sankey et al.^23^ showed no significant effect of fire on most soil properties, including SOC, suggesting soils in this region are relatively resilient to fire. However, there was no apparent consideration of landscape controls on fire influencing these microsite effects such that questions remain about transferability of these findings to complex terrain. One of the few experimental studies examining prescribed fire effects on C fluxes in a more mesic shrubland ecosystem documented rapid recovery of gross ecosystem production (GEP) and net carbon uptake as measured by net ecosystem exchange, supporting the hypothesis that higher elevation, more mesic sites are more resilient to fire disturbance^24^. Vegetation surveys and remote sensing supported rapid recovery of primary productivity associated with growth of the grass community immediately after fire. However, Fellows et al.^24^ also showed little to marginal responses in terms of ecosystem respiration following prescribed fire. This apparent lack of respiratory pulse after disturbance appears to be at odds with ecological theory. This finding indicates a need to revisit and test these models in non-forested ecosystems and possibly incorporate interactions across scales to adequately represent them.

**Figure 2:**
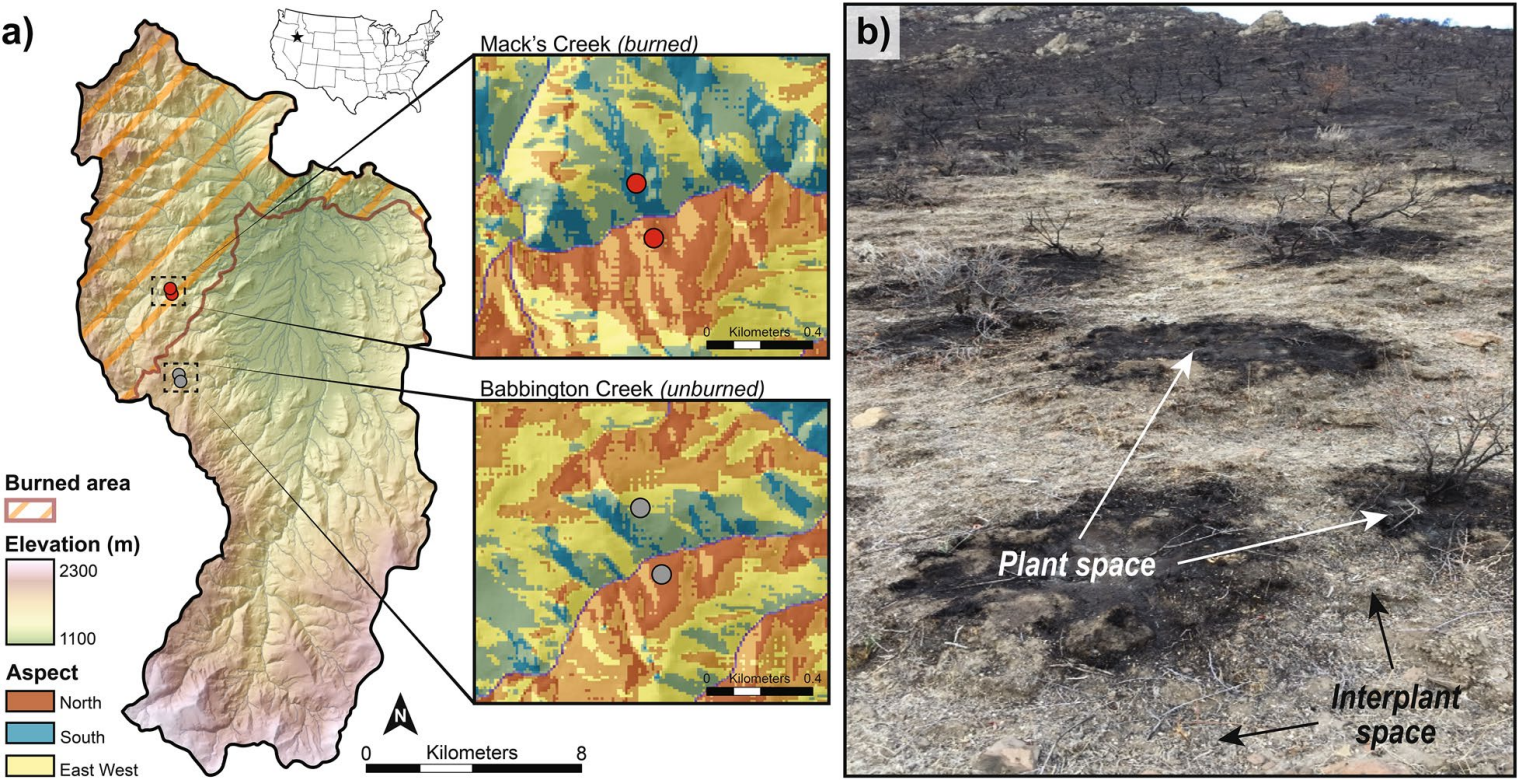
Aspect and microsite properties contrast in study catchments at the Reynolds Creek Experimental Watershed-Critical Zone Observatory (RCEW-CZO) in Southwestern Idaho, USA. (**a**) North- and south-facing aspects within Mack’s Creek (burned) and Babbington Creek (unburned) in the RCEW-CZO. (**b**) Image highlights burned biomass and remanent plant-interplant spaces after the Soda fire in August 2015.

Here we used a state factor approach to evaluate the response and recovery of sagebrush shrublands to a wildfire event across a range of scales at the Reynolds Creek Experimental Watershed and Critical Zone Observatory (RCEW-CZO) in southwestern Idaho. Specifically, we selected two catchments, one that had been burned in the Soda Fire in August 2015 and one that had remained unburned, holding other state factors such as granite parent material, climate, topography and vegetation relatively constant. In each of these catchments, we measured soil surface (to 2 cm depth) properties and processes at four paired plant-interplant locations on north- and south-facing aspects at 2, 3, 6, and 37 months after fire (Fig. 2b). Aboveground biomass was largely eliminated by the fire (Fig. 2a). We hypothesized that soil pH would increase, SOC would decrease, and pyrogenic carbon (PyC) would increase following fire disturbance, but that these patterns would be aspect, microsite, and time dependent. Based on Chambers et al.^20^, we expected north-facing aspects with higher SOC to be more resilient to fire and lose proportionally less carbon than south-facing ones and recover faster following fire. We also expected under plant microsites to have higher SOC than interplant ones and therefore be more resilient to fire. Finally, we expected a marginal respiration pulse immediately following fire, similar to Fellows et al.^24^.

## Results

### Altered soil properties

**Figure 3:**
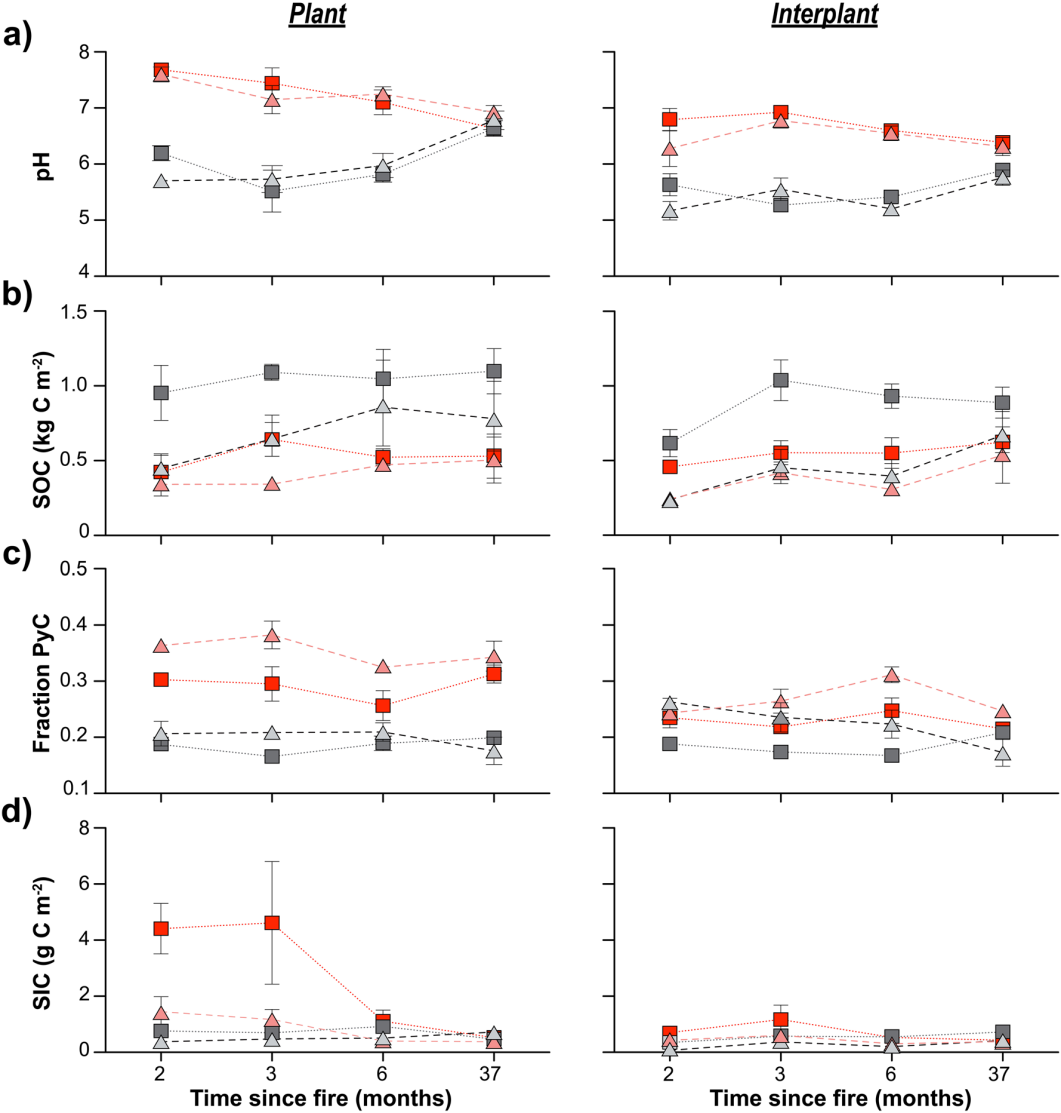
Soil properties exhibit scale-dependent responses and recoveries after fire. Comparison of (**a**) soil pH, (**b**) soil organic carbon (SOC), (**c**) fraction of pyrogenic carbon (PyC), and (**d**) soil inorganic carbon (SIC) on burned (red and pink) and control (dark and light grey) north- (squares) and south-facing aspects (triangles) 2–37 months after fire.

Consistent with expectations, soil pH increased significantly, SOC decreased, and PyC increased following fire, but the burn effects often varied with aspect and/or microsite (Fig. 3). In the case of soil pH, only a main burn effect was observed with average soil pH increasing by an order of magnitude on burned sites immediately after fire compared to controls (burn: F_1,12_ 374, p < 0.0001), 7.08 ± 0.11 compared to 5.67 ± 0.17 (Supplementary Table S1). No microsite effect was observed for soil pH based on repeated measures multivariate analysis of variance (RMANOVA) test on paired plant-interplant differences (p = 0.562) (*see Statistical Analyses in Methods for more detail*). In addition, there was no significant difference in soil pH between north- and south-facing aspects (p = 0.214) and no burn-aspect interaction (p = 0.8140). Soil pH decreased significantly with time in the burn treatments (F_3,10_ 22.81, p < 0.001) and returned to control levels under previous plant canopies within 37 months. Burned interplant spaces remained significantly elevated after 37 months, regardless of aspect (p < 0.05) (Fig. 3a).

SOC decreased significantly following fire, but the patterns were aspect and microsite dependent. In contrast to pH, a significant microsite effect was identified based on a RMANOVA performed on paired plant-interplant differences (F_1,12_ 6.34, p = 0.027) (Fig. 3b, Supplementary Table S1). Furthermore, RMANOVA tests on plant-specific SOC stocks showed that average SOC stocks under previous plants on the burned aspects were significantly lower compared to unburned controls (F_1,12_ 25.4, p = 0.003), with a significant aspect (F_1,12_ 9.46, p = 0.0096) but no significant burn-aspect, time, and interaction effects (Fig. 3b). Indeed, SOC stocks under previous plants on burned north-facing aspects were reduced by half 2 months after fire, 0.42 ± 0.011 kg C m^−2^ on north burn compared to 0.96 ± 0.018 kg C m^−2^ on north control. This contrasted with burned south-facing aspects where SOC stocks were reduced but were not significantly different than south controls, 0.34 ± 0.077 compared to 0.448 ± 0.010 kg C m^−2^ (p < 0.05). For interplant microsites, SOC decreased significantly with burn, aspect, and its interaction (burn: F_1,12_ 11.29, p = 0.005, aspect: 27.19, p = 0.0002, burn x aspect: 5.08, p = 0.04, and time: 5.04, p = 0.02) (Fig. 3b). Thirty-seven months post-fire, SOC stocks under previous plants remained significantly lower on burned compared to control (p < 0.05), especially on north-facing aspects, whereas SOC stocks in interplant spaces on both burned aspects were not significantly different from controls.

### Emergent scale dependent soil properties

While SOC stocks decreased more on north- than south-facing aspects after fire, the fraction of SOC as PyC increased significantly under previous plant canopies, most notably on south-facing aspects (burn: F_1,12_ 201.28, p < 0.0001, aspect: 17.37, p = 0.0013, burn × aspect, 6.55, p = 0.025) (Fig. 3c). Post-hoc Tukey tests showed that mean PyC fraction on burned south-facing aspects was significantly higher than on burned north-facing aspects, 0.36 ± 0.012 compared to 0.30 ± 0.006, which were significantly higher than both controls (0.187–0.206) (p < 0.05). The increase in PyC was more modest but also significant in interplant spaces (burn: F_12_ 20.02, 0.008, aspect: F_12_ 15.49, 0.002). PyC did not vary with time and remained significantly elevated under previous plants and interplant spaces.

**Figure 4:**
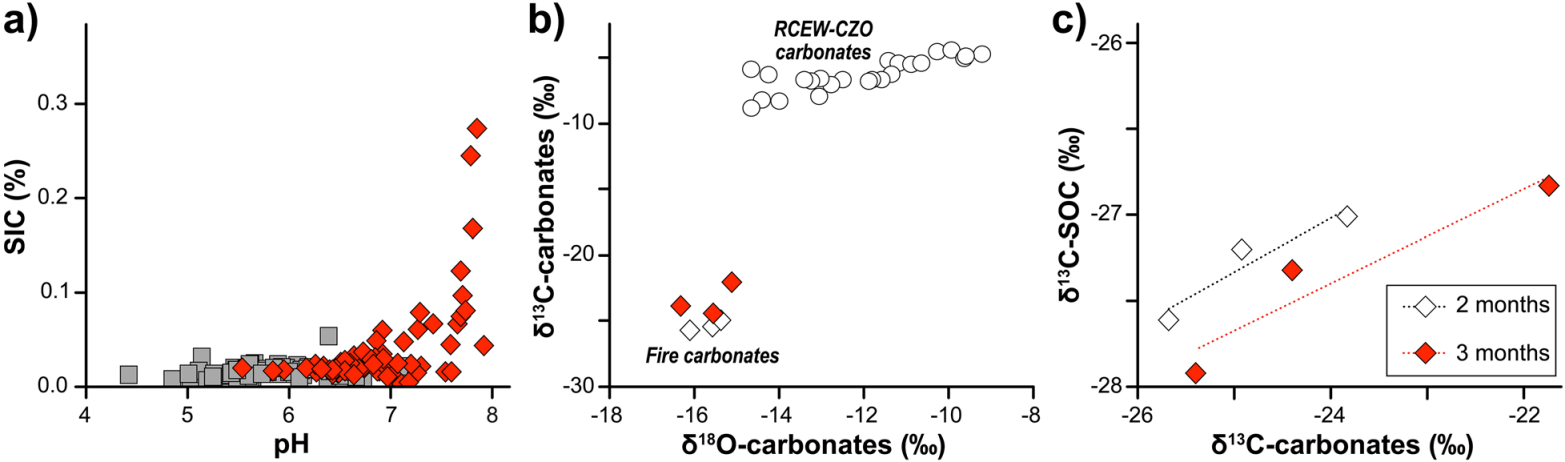
Soil pH and carbonate isotopes values provide possible mechanisms for SIC formation. (**a**) A threshold associated with soil inorganic carbon (SIC) formation was observed as pH increases following fire, burn soils (red), control (grey), (**b**) Plot of δ^13^C- and δ^18^O-carbonate values depicting depleted fire carbonate isotope ratios relative to observed soil carbonates at depth in the RCEW-CZO, and (**c**) the correlation of δ^13^C-carbonate values with δ^13^C-SOC values.

In addition to PyC increases, we found that soil inorganic carbon (SIC) formed in largely carbonate-free soils either during or immediately after fire (Fig. 3d). Specifically, SIC increased significantly and preferentially under previous plants compared to interplant spaces, and it differed significantly with aspect, with significantly more SIC on north- than south-facing aspects (burn: F_1,12_ 31.61, p < 0.0001, aspect: 24.51, p = 0.003, burn-aspect, 16.08, p = 0.0017). 2 months after fire, SIC stocks in plant microsites were as high as 4.41 ± 0.90 g C m^−2^ and 1.4 ± 0.54 g C m^−2^ on burned north- and south-facing aspects, respectively. SIC was only marginally significantly elevated in burned interplant microsites (burn: F_1,12_ 4.38, p < 0.058, aspect: 13.3, p = 0.003, burn-aspect, 0.0003, p = 0.98) (Fig. 3d). SIC in plant microsites decreased significantly with time since fire and returned to background levels within 37 months (F_3,10_ time: 17.92, p = 0.003, time x burn: F_3,10_ 21.17, p = 0.0001, time × aspect: F_3,10_ 7.63 p = 0.006, time × burn × aspect: F_3,10_ 5.67, p = 0.015). SIC appeared to form above a pH threshold of 6.5 (Fig. 4a). Post-fire carbonates (with enough mass to be run for isotopes, n = 6) revealed that the carbonate isotopes were markedly depleted in both δ^13^C and δ^18^O values compared to RCEW-CZO pedogenic soil carbonates formed at depth (Fig. 4b). Post -fire δ^13^C-carbonate values were correlated with SOC-δ^13^C values (Fig. 4c).

### Marginal respiratory losses

**Figure 5:**
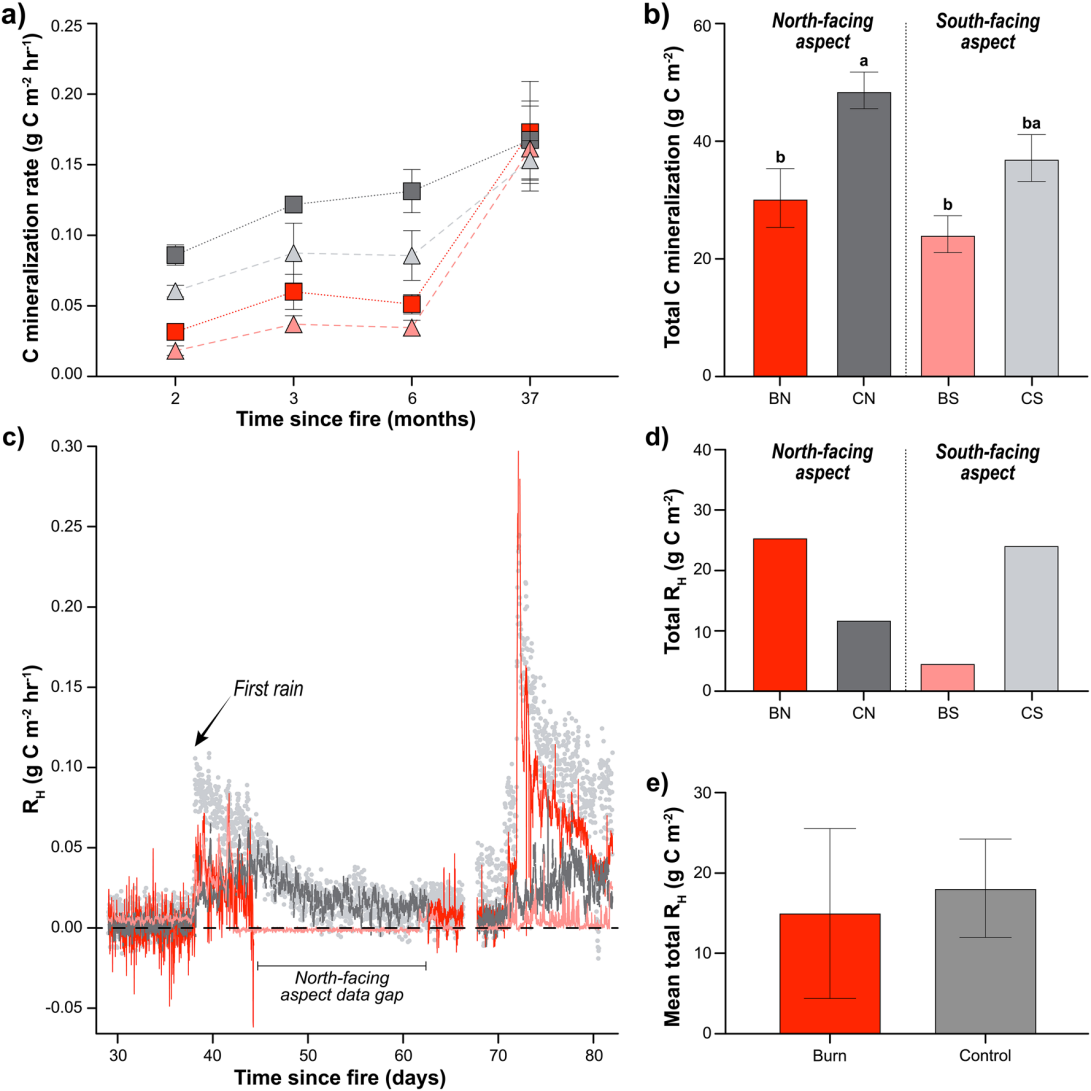
Respiration on burned aspects was reduced relative to controls in both lab and field measurements. (**a**) Soil carbon (C) mineralization rates with time (months) since fire on burned (red and pink) and control (dark and light gray) for north- (squares) and south-facing aspects (triangles) 2–37 months after fire, (**b**) total C mineralization that occurred during this period, (**c**) respiratory fluxes (R_H_) with reduced and even negative fluxes immediately after fire in September (~ 30 days post-fire) on the burned north-facing aspect (red line), (**d**) total losses from each site showing variable losses from different burned and control aspects, and (**e**) average total fluxes from burned and control aspects were not significantly different owing to difference in aspect.

We evaluated respiratory losses (R_H_) (Fig. 1) using multiple field approaches to capture CO_2_ effluxes along with controlled laboratory incubations to evaluate potential C mineralization. All approaches generally showed reduced and aspect-dependent CO_2_ effluxes immediately after fire. Consistent with expectations, rates of potential C mineralization were significantly reduced on burn aspects relative to controls immediately after fire, with marginal aspect and no microsite effects (F_1,12_ burn: 12.25 p = 0.004, aspect: 4.42, p = 0.057) (Fig. 5, Supplementary Table 1). Carbon mineralization rates varied significantly with time (F_3,10_ 20.43, p = 0.0001) and returned to control levels within 37 months (Fig. 5a). Cumulative C mineralization losses on burned north and south aspects were significantly lower than north controls but not the south controls (p < 0.05) (Fig. 5b). In line with these observations, continuous field CO_2_ fluxes measured by forced diffusion (FD) chambers (Eosense, Dartmouth, NS, CAN) showed fluxes from burn aspects were similar or lower (and even negative) compared to controls immediately following fire (within a month of fire containment) (Fig. 5c). A respiratory pulse was observed from all sites after the first rains in October, with larger total R_H_ losses from the burned north-facing aspect than its control, although losses were comparable to the south control (Fig. 5d). Average total R_H_ losses from the burn aspects were reduced but not significantly different than controls (Fig. 5e).

## Discussion

**Figure 6:**
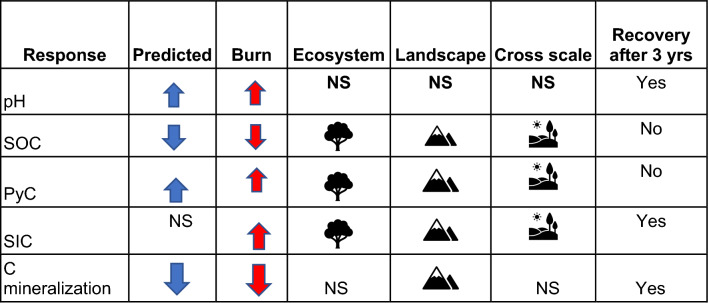
Cross-scale responses to fire were observed. Observed main burn response compared to predicted response based on disturbance theory and Fellows et al. ^24^ in the case of C mineralization. We found ecosystem and landscape scale dependent responses and interactions across different scales. Recovery after 3-year post-fire (yes or no) is reported showing that recovery varies with soil property and process.

We found significant changes in soil properties and processes following fire, some of which varied with landscape- and ecosystem- scale and their cross-scale interactions. Others such as PyC and SIC were emergent properties following fire. Some of these properties and processes recovered and returned to ambient conditions within 37 months, while others did not (Fig. 6). We describe these changes in scale dependent structure and function and explore possible mechanisms explaining observed SIC formation.

### Changes in scale dependent structures

Consistent with our expectations, we found that soil pH increased significantly following fire. In contrast to our expectations, the burn effects on pH were not shrub microsite or aspect scale dependent (Figs. 3a, 6). Meanwhile, SOC pools were significantly affected by burn, aspect, microsite, and their interaction. These findings contrast with a meta-analysis conducted by Sankey et al.^23^ showing no significant shrub microsite effect on SOC pools. We also found that emergent properties, PyC and SIC, were strongly aspect and microsite-scale dependent (Fig. 3c). Differences in fire severity and/or lower initial stocks of SOC on the south aspects may explain the higher fraction of PyC formed under previous plants on the south-facing aspects. PyC also persisted in plant sites after 37 months suggesting a structural shift in the SOC composition. While Santin et al.^4^ have incorporated PyC into landscape recovery dynamics, our findings point to contrasts in PyC accumulation at the plant-interplant scale that as of yet, have received little to no attention in models of nonforested systems. Finally, in contrast to PyC, SIC formed preferentially on north-facing aspects under former shrubs. To our knowledge, this scale dependent formation of SIC structure after fire has not been quantified, and we expand on this more below. These conflicting results showing microsite effects might in part be explained by the fact that landscape position was considered in this study. In addition, sampling methodology differences (timing of sampling, depth of sampling) could help to explain differences in microsite responses to fire as the ability to detect change in soils collected 0–15 cm was likely lower compared to shallow surface soils (0–2.5 cm) in this study. These results raise questions about previously assumed microsite responses and highlight the need to consider landscape-level processes. Together, these findings showing ecosystem to landscape scale dependent responses in ecosystem structure indicate that theoretical disturbance models need to be revisited to incorporate these multiscale responses.

### Recovery time following disturbance

Based on Chambers et al.^20^, we expected north-facing aspects with higher SOC to be more resilient and recover faster following fire. While soil pH mostly recovered on both aspects and microsites within 37 months of fire, SOC stocks were slower to recover under previous plants, especially on the north-facing aspect (Fig. 3b). This slower recovery on north-facing aspects may in part reflect preferential wind erosion and redistribution of surficial organic matter and fine fraction on north-facing slopes into swales and then subsequent loss of this material via water erosion^25^. It is also likely that reduction of soil cohesion after fire would have preferentially affected the north-facing aspect because it has finer textured soils^16^ and steeper gradients than the south-facing slopes^17,18^. Our results provide little support that soils on north-facing aspects are more resilient to disturbance relative to soils on south-facing aspects as predicted by Chambers et al.^20^.

### Marginal respiration pulse

A large R_H_ pulse was not observed following fire (Fig. 1). Consistent with Fellows et al.^24^, we found lower rates of respiration immediately after fire in both incubation and field studies. Rates of potential C mineralization were significantly reduced on both burn aspects, with marginal aspect and no microsite effects. Rates returned to ambient levels within 37 months indicating the more soluble carbon pools were able to recover, while other pools were not. Field R_H_ was more variable (Fig. 5d), but average total R_H_ after fire was comparable to unburned R_H_ (Fig. 5e). Interestingly, R_H_ was low and even negative on the north-facing control and burn aspects indicating consumption of carbon dioxide^26^. Below, we suggest a possible pathway of carbon storage in drylands after fire that is not currently represented in theoretical models (Figs. 1, 6).

### Possible mechanisms contributing to carbonate formation

We found preferential and ephemeral formation of soil carbonates under previous plant canopies on burn north-facing compared to south-facing aspects and no carbonate formation in interplant microsites. Previous studies at the RCEW-CZO have shown that pedogenic carbonate formation is restricted to sites with annual precipitation less than 500 mm^13,27,28^, yet these catchments were above this threshold (550–600 mm) and little to no carbonate was observed previously^13^. Further examination of the controls on carbonate formation showed that carbonates increased nonlinearly as a function of soil pH (Fig. 4a) and that post-fire carbonate isotopes were markedly depleted in both δ^13^C and δ^18^O compared to pedogenic soil carbonates formed at depth at the RCEW-CZO (Fig. 4b). Furthermore, the formation of SIC under plants compared to the marginal formation in interplant spaces provided clues as to the possible mechanisms allowing for the formation of carbonates under some post burn conditions.

Equilibrium theory indicates that carbonates can form when solution becomes supersaturated with calcite^29^ where

1 $${\text{CaCO}}_{{3}} + {\text{ CO}}_{{2}} + {\text{ H}}_{{2}} {\text{O }} = {\text{ Ca}}^{{{2} + }} + {\text{ 2 HCO}}_{{3}}^{ - } .$$

Possible mechanisms explaining carbonate formation include a decrease in partial pressure of CO_2_ (pCO_2_) in the soil or an increase in the relative concentrations and activities of dissolved calcium [a(Ca^+2^)] and bicarbonate ([a(HCO_3_^−^)]^2^, possibly due to decreased water content where

2 $${\text{K}}_{{{\text{cal}}}} = \, \left[ {{\text{a}}\left( {{\text{Ca}}^{{ + {2}}} } \right)} \right] \, \left[ {{\text{a}}\left( {{\text{HCO}}_{{3}}^{ - } } \right)} \right]^{{2}} {/ }\left[ {{\text{pCO}}_{{2}} } \right]$$

such that K_cal_ is the equilibrium constant for calcite.

The relative distribution of dissolved inorganic carbon species is mainly controlled by pH and pCO_2_ where the concentration ratio [CO_3_^2–^]/[CO_2_] is a function of the [H^+^]. Thus, higher pH following fire as observed in Figs. 3 and 4 apparently favored the formation of HCO_3_^-^ and carbonates as SIC (Fig. 4). Calcium previously stored in plant biomass would have also been made available as ash in the soil and promoted formation of carbonates under canopies compared to interplant spaces (Figs. 2, 4a, b). Finally, a reduction in carbon mineralization or respiration and decrease pCO_2_ post fire, both of which were observed in lab and field, could further promote the formation of carbonate.

Carbonate isotopes also provided a clue about their origin. δ^13^C-carbonate values of burned soils were similar to the δ^13^C of CO_2_ of biogenic origin whereas δ^18^O values resembled groundwater or cold-season precipitation inputs^30^. It is typical that soil carbonate formation results in a systematic enrichment of 13.5–16.5 δ^13^C relative to the δ^13^C of respired CO_2_^31,32^, both as a function of phase transformation from CO_2_ to CaCO_3_, and preferential ^12^CO_2_ diffusion. δ^13^C values of pedogenic carbonates derived solely from respired CO_2_ would range between − 13.6 and − 7.6 ‰, or with an equal mixture with atmospheric CO_2_ closer to − 4.0 ‰. Thus, the unexpected depletion of post-fire carbonate-δ^13^C values represents a potential unaccounted for carbon fractionation mechanism post fire. Other mechanisms explaining the formation of carbonates includes formation from combustion of calcium oxalates in stems, leaves, and roots under lower temperatures < 500 °C and the hydration of calcium oxide abiotically to calcium carbonate or bicarbonate^33^. However, these mechanisms would not induce depletion of carbonate-δ^13^C values (Fig. 4b). Similarly, complete consumption of the SOC and conversion of all the SOC-sourced CO_2_ to HCO_3_^-^ and subsequently SIC still would not result in depletion of carbonate-δ^13^C values to the degree observed here.

A possible explanation for such carbonate isotope values is an extracellular oxidative metabolism (EXOMET)^34^. EXOMET is C mineralization devoid of active biological metabolism via soil-stabilized enzymes or soil mineral, metal, and PyC catalysts, and known to induce a strong carbon isotope fractionation in evolved CO_2_ (≤ 50 ‰) for several months. Evidence for EXOMET activity in burn soil contributing to carbonate formation immediately after fire includes the correlation (r^2^ = 0.9) between δ ^13^C-SOC and depletion of carbonate-δ ^13^C values (Fig. 4c). Our results collectively indicate that a combination of conditions, high fire temperature under shrubs, increases in soil pH, a reduction in respiration and decrease pCO_2_, and increase in the availability of calcium from ash were likely mechanisms resulting in the formation of carbonates and that EXOMET activity might have catalyzed this formation. Together, these findings indicate a previously overlooked, short-term sink or storage of carbon as SIC on the landscape, followed by an additional medium-term C loss pathway. Further experimental study of SIC formation after fire beyond these field observations is merited to understand the formation and fate of carbonates. The scope of our findings is limited given that our samples only represented shallow soil depths and two watersheds in the RCEW-CZO in Idaho, though we note that Goforth et al.^35^ documented effervescence after a wildfire in the San Gabriel Mountains suggesting the formation of carbonates elsewhere after fire. We anticipate that the controls on the proposed pathway of post-fire SIC formation to further depend on fire intensity, soil climate, and physical and chemical properties.

Other loss pathways not considered in this study but documented in other studies such as wind and water erosional losses may also need consideration in dryland disturbance models. In particular, large losses as sediment and associated particular organic carbon were documented in the two years following the fire, a total of 1.17 and 0.0625 kg m^−2^ sediment and carbon, respectively^36^. These losses were an order of magnitude higher than unburned catchments^36^. Vega et al.^25^ further showed that the fire response was a two-step process with wind erosion loading leeward (north) aspects preferentially in swales, and these swales were emptied to streams by water erosion^36^. These results again underscore the importance of incorporating multi-scale domains into disturbance models in these regions.

In conclusion, our findings confirm that these non-forested ecosystems do not respond with a large respiratory pulse immediately after disturbance as found in non-forested ecosystems; rather they appear to form short-term carbonate structures preferentially under shrubs. In contrast to our expectations and others, we found little evidence of north-facing aspects being more resilient than south-facing ones as north facing aspects lost proportionally more SOC than south facing ones, and both aspects did not recover back to ambient SOC levels. While fire significantly reduced mineralizable C, these processes recovered within 37 months. PyC under burned plants appears to be a persistent shift in the structural component of SOC that merits more study, particularly in regards to ecosystem resilience. We conclude that non-forested systems have multi-scale structure and functions, and conditions during and following fire result in cross-scale interactions that amplify some structures but not others that are not represented in current theoretical disturbance models.

## Methods

### Study site

This study was conducted at the Reynolds Creek Experimental Watershed and Critical Zone Observatory (RCEW-CZO), a semiarid catchment representative of Intermountain West United States^37^. Located in southwestern Idaho, the RCEW (239 km^2^) extends over a steep climatic gradient (mean annual precipitation 250—1100 mm year^−1^, mean annual temperature 5.5–11 °C) (Fig. 2). The environmental variability is driven by the nearly 1000 m elevation range and contains variable geology as described below. Rain is the dominant form of precipitation in the RCEW, with snow dominating in the highest elevations. Corresponding vegetation types include Wyoming big sagebrush steppe (*Artemesia tridentata wyomingensis*) in the lower elevations, transitioning to mountain sagebrush (*Artemesia tridentata *ssp. *vaseyana*)*,* western juniper (*Juniperus occidentalis*), aspen (*Populus tremuloides*)) and Douglas Fir (*Pseudotsuga menziesii*) at higher elevations. The geology of the Reynolds Creek basin consists of Neogene volcanic and sedimentary rocks overlying Cretaceous granitic basement^38^.

Starting August 10, 2015, the Soda Fire burned 113,000 hectares (279,000 acres) of sagebrush steppe habitat in southwestern Idaho and was contained by August 22, 2015. The fire severity was rated predominately moderate severity (yellow) in the areas of study with pockets of high severity (red) (USGS image source, Landsat 7)^39^. Approximately 29% or 68 km^2^ of the RCEW-CZO was burned. Given the focus of the RCEW-CZO on improving prediction and understanding of soil carbon at the pedon to landscape scale^37^, the Soda Fire provided a unique opportunity to examine how fire alters soil carbon stocks and its recovery.

### Experimental design

Two catchments, Babbington Creek and Mack’s Creek, were selected for study (Fig. 2). Mack’s Creek was located within the burn perimeter of the Soda Fire, and Babbington Creek was selected as the control due to its similarity to Mack’s Creek (Fig. 2). Specifically, catchments were selected such that state factors (i.e. lithology, vegetation, topography, and climate) were held relatively constant^40^. Both catchments were underlain by granodiorite lithology^38^ and experienced a mean annual precipitation of 640 mm and a mean annual temperature of 6.6 °C based on available climate data^41^. Elevation locations were selected to match climate. Catchments have predominantly north- and south-facing aspects (Fig. 2) with Wyoming Big Sagebrush (*Artemisia tridentata* ssp. *wyomingensis*) as the dominant plant species on both aspects representing 50–75% and bitterbrush (*Purshia stansburyana*), grasses, and western juniper (*Juniperus occidentalis*) making up the remaining major vegetation.

Sites were established on the north- and south-facing aspect slopes using a wheel-and-axle sampling design. Each aspect contained four shrub sites with paired interplant space (microsite). Soil sample collection occurred 2, 3, and 6, and 37 months after the fire. Surface samples were collected from 0 to 2.5 cm depth. Others at 2.5–5, 5–10, and 10–20 cm were also collected but not the focus of this study. The fine fraction (< 2 mm) was separated from the coarse fraction (> 2 mm) to provide percent coarse fraction by weight. Soils were analyzed for pH, SOC, pyrogenic carbon (PyC), and SIC.

### Soil methods

#### Soil pH

Soil pH was determined following standard methods^42^. Briefly, we determine soil pH on 1:1 soil to 18.2 Mohm water slurry paste after 1 h to allow equilibration. The pH meter was calibrated using the pH 4, 7, and 10 buffers and a check calibrant was checked every 10 samples and accepted within 0.02 units.

#### SOC and stable isotopes

SOC and isotopes were determined on an Elemental Analysis-Isotope Ratio Mass Spectrometry (EA-IRMS) at Idaho State University’s Center for Archaeology, Materials and Applied Spectroscopy (CAMAS) laboratory. Soils were pretreated with 5% v/v hydrochloric acid (HCl) to remove soil inorganic carbon^43^, ball-milled, and dried prior to packing. Carbon stocks (kg m^−2^) were estimated from bulk density using Patton et al.^44^ adjusting for measured differences in coarse fraction^44^.

#### Pyrogenic carbon (PyC)

Pyrogenic carbon (PyC) was estimated using a chemometric model relating Fourier-transform mid-infrared spectra of the soil samples to lab-based estimates of PyrC content as described in McGuire et al.^45^. PyC are compounds produced from incomplete combustion and include soot, charcoal, biochar, black carbon, elemental carbon^46^. The training set consisted of 287 samples from Australia^47^ and 99 samples from the United States^48^ that underwent the same carbon fractionation procedure^47^ which isolates particulate, mineral-associated and pyrogenic carbon fractions using a combination of physical size fractionation and solid-state ^13^C nuclear magnetic resonance (NMR) spectroscopy. Finely milled subsamples of the same samples along with all of the samples in this present study were scanned on a Bruker Vertex 70 FTIR with a Pike Autodiff diffuse reflectance accessory. Spectra were acquired in the range of 6000–180 cm^−1^ at a 4 cm^−1^ resolution. A partial least squares regression model built on standard normal variate transformed spectra could explain 86% of the variation in observed PyC data with a root mean square error of 0.32 g kg^−1^^45^.

#### Soil inorganic carbon (SIC)

Soil inorganic carbon (SIC) was measured initially using effervescence tests with a 1 N HCl drops. The expectation was that there would be little to no effervescence given the lack of it prior to the fire. However, tests revealed moderate to high effervescence, which warranted further testing on the samples in order to quantify inorganic carbon by modified pressure-calcimeter method^49^. In brief, 1 g soil or standard was added to a 20 mL or 100 mL serum vial and 2 mL of 6 M hydrochloric acid (HCl) with 3% by weight ferrous chloride tetrahydrate chloride (FeCl_2_∙4H_2_O) was added in a dram and sealed with a Wheaton rubber stoppers, (no. 224100-192) and aluminum cap and crimper. The bottles were agitated to release the acid and allowed to sit overnight before measurement on a pressure transducer (Setra model 280, 0–15 PSIG, 0.03–5.03 VDC output). Six standards containing incrementally increasing calcium carbonate (CaCO_3_) in a sand mesh matrix. CaCO_3_ was converted to SIC using the following

3 $$\% {\text{SIC }} = \, \% {\text{ CaCO}}_{{3}} *{ 12}/{1}00.$$

Finally, a select set of samples were run for carbonate isotopes and compared to samples analyzed at a select set of sites.

#### Carbonate isotopes

Isotopic analysis of SIC pools was done separately from analysis of SIC pools and involved removing organic material from air-dried soils by adding 10 mL of 5% sodium hypochlorite to each sample and agitating for 24 h^50^. Following digestion, each sample was centrifuged for 5 min at 3600 rpm to settle clay particles, and then decanted. To remove trace amounts of sodium hypochlorite, samples were rinsed with 10 mL of deionized water, and the centrifuging-decanting process repeated twice. Soil samples were then freeze-dried prior to analysis at CAMAS. Samples were analyzed on a GasBench II. Analysis consisted of purging 12 mL screw top Exetainer™ sample vials using helium and subsequent acidification with 0.2 mL of 19.3 M phosphoric acid (H_3_PO_4_), volatilizing SIC as CO_2_. The evolved CO_2_ was routed through a ThermoScientific Delta Advantage stable isotope ratio mass spectrometer, as above. The ratio of ^13^C to ^12^C is reported as δ^13^C using standard delta notation. Isotopic ratios from SOC pools were used to constrain source pool for SIC-δ^13^C values. Carbon values determined by calcite volatilization were compared to molar C content determined by the calcimeter method to assess losses of calcite to SOC removal treatments; change in percent SIC-C was not significant between methods.

#### Field measured carbon dioxide efflux

We monitored field carbon dioxide efflux using four automated forced diffusion (FD) chambers (Eosense, Dartmouth, NS, CAN) that were deployed within one month of the fire on north and south facing aspects on burned and unburned catchments. The chambers operate similarly to surface chambers, but require no flow or flushing of atmospheric air. Instead, they measured purely diffusive transport by monitoring concentration gradients across membranes of known permeability and can monitor CO_2_ efflux every 15 min^51^. Given gaps in data, we summed fluxes where all four chambers had data present during the study period. Owing to major challenges with access (16 km roundtrip) and maintaining chamber power and stability on burned hillslopes in winter, we removed the chambers.

#### Potential soil carbon mineralization

We compared these field fluxes to potential rates of carbon mineralization on air-dried soils following Fierer and Schimel^52^. While a less conventional approach, air-dried soils provided the opportunity to control for environmental conditions and moisture content and evaluate the potential or relative rates of carbon mineralization following fire. Carbon mineralization rates were measured regularly once a day over a 4 day period. In brief, we added 20 g of fine-fraction (< 2 mm) soil into 235 mL mason jars and brought the soils up to 60% water filled pore space, based on prior determination of water holding capacity^53^. Jars were sealed with a rubber septa in the lid at the start of the incubation, and 5 mL gas sample was drawn at time zero (t_0_) and after 4 h (t_f_), and injected into a EMG 4 (PP Systems, Amesbury, MA) infrared gas analyzer (IRGA). Temperature and barometric pressure were also monitored. Between the daily 4-h snap shot incubations, lids were removed from the jars, jars were vented to the atmosphere, jars were covered with saran wrap to allow to exchange gas with atmosphere but minimize water loss and sealed with rubber bands, and stored in the dark. Carbon mineralization (ug C g dry soil^−1^ h^1^) was calculated from the difference in concentrations and divided by the g dry soil and incubation time (4 h). We determine the average rate of carbon mineralization based on the mean of the 4 daily snap shot incubations. We converted these values g C m^−2^ h^−1^ using Patton et al.^44^ as described above. We also calculated cumulative carbon mineralized by multiplying the hourly rates by 24 and summing the total losses (ug g dry soil^−1^) and converted these an areal basis (g C m^−2^).

#### Statistical analyses

Given the balanced, repeated, paired plant-interplant design, we first tested for influences of burn (B), aspect (A), and its interaction (B*A) on plant-interspace properties by conducting a Repeated measures multivariate analysis of variance (RMANOVA) in JMP Pro 16 (Carey, NJ) on the difference between the pairs. If no significant differences were detected, we pooled the plant and interplant data and averaged it and then performed a RMANOVA, to retain correct degrees of freedom. If there were significant effects based on plant-interplant differences, we conducted a RMANOVA on separate plant or interplant properties.

## Supplementary Information

Supplementary Table S1.

## Data Availability

Data are available at BSU ScholarWorks Reynolds Creek Critical Zone Observatory (10.18122/reynoldscreek.28.boisestate).
