# Supplementary material for: Multiscale responses and recovery of soils to wildfire in a sagebrush steppe ecosystem
Source: Sci Rep. 2022 Dec 23;12:22438. doi: 10.1038/s41598-022-26849-w (PMC9794722; doi:10.1038/s41598-022-26849-w)
Supplement: Supplementary file 1 — Supplementary Table S1. [file 41598_2022_26849_MOESM1_ESM.pdf]

**Scale-dependent responses and recovery to wildfire in non-forested shrubland ecosystems**

Kathleen A. Lohse[1,2], Derek Pierson[1], Nicholas R. Patton[2], Jonathan Sanderman[3], David P. Huber[1], Bruce Finney[1,2], Jeremy Facer[1], Jared Meyers[1], Mark S. Seyfried[4]
1 Department of Biological Sciences, Idaho State University, Pocatello, ID
2 Department of Geosciences, Idaho State University, Pocatello, ID
3 Woodwell Climate Research Center, Falmouth, MA
4 Northwest Watershed Research Center, USDA ARS, Boise, ID

**Supplementary Table S1:** Repeated Measures ANOVA tests for soil properties including soil pH, soil organic carbon (SOC), Pyrogenic carbon (PyC), soil inorganic carbon (SIC), and cumulative mineralization (Cum Min) rates. Paired plant-interplant (PIP) were subtracted from each other to obtain a PIP difference (PIP-diff). A RMANOVA was performed to evaluate if there was a PIP or shrub microsite effect. If no shrub microsite effect was detected, then PIP pairs were pooled, averaged (PIP-avg) and RMANOVA performed on pooled data for effects of burn, aspect, and time and their interaction terms. If a PIP effect was detected, then RMANOVAs were performed on plant and interplant microsites separately (-P or -IP). Values (p>F) are considered significant when p<0.05 and bolded. Degrees of freedom (DF) for numerator (n) and denominator (d) are shown and exact F value is reported.

| Response Variable | Factor | DF (n, d) | Exact F | Prob>F |
|---|---|---|---|---|
| Soil pH PIP-diff | | | | |
| | Burn | 1,12 | 0.354 | 0.562 |
| | Aspect | 1,12 | 1.399 | 0.2597 |
| | Burn*Aspect | 1,12 | 0.067 | 0.798 |
| | Time | 3,10 | 1.99 | 0.179 |
| Soil pH PIP avg | | | | |
| | **Burn** | 1,12 | 374.78 | **<0.0001** |
| | Aspect | 1,12 | 1.72 | 0.2141 |
| | Burn*Aspect | 1,12 | 0.0602 | 0.8104 |
| | Time | 3,10 | 1.98 | 0.179 |
| | **Time*Burn** | 3,10 | 22.89 | **<0.0001** |
| | Time*Aspect | 3,10 | 2.815 | 0.0937 |
| | Time*Burn*Aspect | 3,10 | 1.11 | 0.389 |

| **Response Variable** | **Factor** | **DF (n, d)** | **Exact F** | **Prob>F** |
|---|---|---|---|---|
| SOC PIP-diff | | | | |
| | **Burn** | 1,12 | 7.86 | **0.0269** |
| | Aspect | 1,12 | 6.348 | 0.4638 |
| | Burn*Aspect | 1,12 | 0.006 | 0.9528 |
| | Time | 3,10 | 0.68 | 0.58 |
| | Time*Burn | 3,10 | 0.28 | 0.84 |
| | Time*Aspect | 3,10 | 0.55 | 0.65 |
| | Time*Burn*Aspect | 3,10 | 0.95 | 0.45 |
| SOC-P | | | | |
| | **Burn** | 1,12 | 25.4 | **0.0003** |
| | **Aspect** | 1,12 | 9.46 | **0.0096** |
| | Burn*Aspect | 1,12 | 2.56 | 0.1356 |
| | Time | 3,10 | 0.909 | 0.4707 |
| | Time*Burn | 3,10 | 0.114 | 0.94 |
| | Time*Aspect | 3,10 | 1.28 | 0.33 |
| | Time*Burn*Aspect | 3,10 | 0.21 | 0.88 |
| SOC -IP | | | | |
| | **Burn** | 1,12 | 11.29 | **0.0057** |
| | **Aspect** | 1,12 | 27.19 | **0.0002** |
| | **Burn*Aspect** | 1,12 | 5.08 | **0.0437** |
| | **Time** | 3,10 | 5.04 | **0.04** |
| | Time*Burn | 3,10 | 0.79 | 0.52 |
| | Time*Aspect | 3,10 | 0.79 | 0.57 |
| | Time*Burn*Aspect | 3,10 | 0.68 | 0.58 |

| **Response Variable** | **Factor** | **DF (n, d)** | **Exact F** | **Prob>F** |
|---|---|---|---|---|
| PyC-PIP diff | | | | |
| | **Burn** | 1,12 | 50.15 | **<0.0001** |
| | **Aspect** | 1,12 | 0.0002 | **0.0013** |
| | **Burn*Aspect** | 1,12 | 3.99 | **0.025** |
| | Time | 3,10 | 2.99 | 0.08 |
| | **Time*Burn** | 8.04 | 0.005 | **0.005** |
| | Time*Aspect | 3,10 | 0.22 | 0.87 |
| | Time*Burn*Aspect | 3,10 | 0.83 | 0.51 |
| PyC-P | | | | |
| | **Burn** | 1,12 | 201.28 | **<0.0001** |
| | **Aspect** | 1,12 | 17.37 | **0.0013** |
| | **Burn*Aspect** | 1,12 | 6.5775 | **0.025** |
| | Time | 3,10 | 1.16 | 0.37 |
| | Time*Burn | 3,10 | 2.47 | 0.12 |
| | Time*Aspect | 3,10 | 1.61 | 0.25 |
| | Time*Burn*Aspect | 3,10 | 0.02 | 0.99 |
| PyC -IP | | | | |
| | **Burn** | 1,12 | 20.02 | **<0.0008** |
| | **Aspect** | 1,12 | 15.49 | **0.0002** |
| | Burn*Aspect | 1,12 | 0.008 | 0.92 |
| | Time | 3,10 | 2.65 | 0.10 |
| | **Time*Burn** | 3,10 | 4.66 | **0.027** |
| | **Time*Aspect** | 3,10 | 4.81 | **0.02** |
| | Time*Burn*Aspect | 3,10 | 2.66 | 0.11 |

| **Response Variable** | **Factor** | **DF (n, d)** | **Exact F** | **Prob>F** |
|---|---|---|---|---|
| SIC-PIP diff | | | | |
| | Burn | 1,12 | 28.22 | **0.0002** |
| | **Aspect** | 1,12 | 15.40 | **0.002** |
| | **Burn*Aspect** | 1,12 | 19.52 | **0.0008** |
| | **Time** | 3,10 | 20.138 | **0.0001** |
| | **Time*Burn** | 3,10 | 16.138 | **0.0004** |
| | **Time*Aspect** | 3,10 | 8.4428 | **0.004** |
| | **Time*Burn*Aspect** | 3,10 | 4.89 | **0.024** |
| SIC-P | | | | |
| | **Burn** | 1,12 | 31.61 | **<0.0001** |
| | **Aspect** | 1,12 | 24.51 | **0.0003** |
| | **Burn*Aspect** | 1,12 | 16.08 | **0.0017** |
| | **Time** | 3,10 | 17.92 | **0.002** |
| | **Time*Burn** | 3,10 | 21.17 | **0.001** |
| | **Time*Aspect** | 3,10 | 7.63 | **0.006** |
| | **Time*Burn*Aspect** | 3,10 | 5.67 | **0.015** |
| SIC -IP | | | | |
| | Burn | 1,12 | 4.38 | 0.058 |
| | **Aspect** | 1,12 | 13.3 | **0.003** |
| | Burn*Aspect | 1,12 | 0.0003 | 0.98 |
| | Time | 3,10 | 1.35 | 0.32 |
| | Time*Burn | 3,10 | 3.67 | **0.05** |
| | Time*Aspect | 3,10 | 0.19 | 0.90 |
| | Time*Burn*Aspect | 3,10 | 0.41 | 0.75 |

| **Response Variable** | **Factor** | **DF (n, d)** | **Exact F** | **Prob>F** |
| --- | --- | --- | --- | --- |
| Cum Min PIP diff | | | | |
| | Burn | 1,12 | 2.45 | 0.562 |
| | Aspect | 1,12 | 0.025 | 0.2597 |
| | Burn*Aspect | 1,12 | 0.14 | 0.798 |
| | **Time** | 3,10 | 22.52 | **<0.0001** |
| | Time*Burn | 3,10 | 1.16 | 0.37 |
| | Time*Aspect | 3,10 | 0.026 | 0.99 |
| | Time*Burn*Aspect | 3,10 | 0.12 | 0.95 |
| Cum Min PIP avg | | | | |
| | **Burn** | 1,12 | 13.69 | **0.003** |
| | **Aspect** | **1,12** | **4.89** | **0.047** |
| | Burn*Aspect | 1,12 | 0.89 | 0.36 |
| | **Time** | 3,10 | 22.69 | **<0.0001** |
| | Time*Burn | 3,10 | 1.34 | 0.31 |
| | Time*Aspect | 3,10 | 0.24 | 0.86 |
| | Time*Burn*Aspect | 3,10 | 0.15 | 0.93 |
